# Proteoglycan 4 Modulates Osteogenic Smooth Muscle Cell Differentiation during Vascular Remodeling and Intimal Calcification

**DOI:** 10.3390/cells10061276

**Published:** 2021-05-21

**Authors:** Till Seime, Asim Cengiz Akbulut, Moritz Lindquist Liljeqvist, Antti Siika, Hong Jin, Greg Winski, Rick H. van Gorp, Eva Karlöf, Mariette Lengquist, Andrew J. Buckler, Malin Kronqvist, Olivia J. Waring, Jan H. N. Lindeman, Erik A. L. Biessen, Lars Maegdefessel, Anton Razuvaev, Leon J. Schurgers, Ulf Hedin, Ljubica Matic

**Affiliations:** 1 Vascular Surgery, Department of Molecular Medicine and Surgery, Karolinska Institutet, 17164 Stockholm, Sweden; till.seime@ki.se (T.S.); moritz.lindquist.liljeqvist@ki.se (M.L.L.); antti.siika@ki.se (A.S.); hong.jin@ki.se (H.J.); eva.karlof@ki.se (E.K.); mariette.lengquist@ki.se (M.L.); andrew.buckler@ki.se (A.J.B.); malin.kronqvist@ki.se (M.K.); anton.razuvaev@ki.se (A.R.); ulf.hedin@ki.se (U.H.); 2 Department of Biochemistry, CARIM, Maastricht University, 6229 ER Maastricht, The Netherlands; a.akbulut@maastrichtuniversity.nl (A.C.A.); rickvangorp@gmail.com (R.H.v.G.); l.schurgers@maastrichtuniversity.nl (L.J.S.); 3 Department of Medicine, Karolinska Institutet, 17164 Stockholm, Sweden; greg.winski@ki.se (G.W.); lars.maegdefessel@ki.se (L.M.); 4 Department of Pathology, CARIM, Maastricht University Medical Center, 6200 MD Maastricht, The Netherlands; olivia.waring@mumc.nl (O.J.W.); erik.biessen@mumc.nl (E.A.L.B.); 5 Department of Surgery, Leiden University Medical Center, 2300 RC Leiden, The Netherlands; lindeman@lumc.nl; 6 Department for Vascular and Endovascular Surgery, Klinikum rechts der Isar, Technische Universität München, 81679 Munich, Germany; 7 Institute of Experimental Medicine and Systems Biology, RWTH Aachen University, 52062 Aachen, Germany

**Keywords:** Proteoglycan 4, smooth muscle cells, atherosclerosis, extracellular matrix, vascular remodeling, calcification

## Abstract

Calcification is a prominent feature of late-stage atherosclerosis, but the mechanisms driving this process are unclear. Using a biobank of carotid endarterectomies, we recently showed that Proteoglycan 4 (PRG4) is a key molecular signature of calcified plaques, expressed in smooth muscle cell (SMC) rich regions. Here, we aimed to unravel the PRG4 role in vascular remodeling and intimal calcification. *PRG4* expression in human carotid endarterectomies correlated with calcification assessed by preoperative computed tomographies. PRG4 localized to SMCs in early intimal thickening, while in advanced lesions it was found in the extracellular matrix, surrounding macro-calcifications. In experimental models, *Prg4* was upregulated in SMCs from partially ligated *ApoE^−/−^* mice and rat carotid intimal hyperplasia, correlating with osteogenic markers and *TGFb1*. Furthermore, PRG4 was enriched in cells positive for chondrogenic marker SOX9 and around plaque calcifications in *ApoE^−/−^* mice on warfarin. In vitro, *PRG4* was induced in SMCs by IFNg, TGFb1 and calcifying medium, while SMC markers were repressed under calcifying conditions. Silencing experiments showed that *PRG4* expression was driven by transcription factors *SMAD3* and *SOX9.* Functionally, the addition of recombinant human PRG4 increased ectopic SMC calcification, while arresting cell migration and proliferation. Mechanistically, it suppressed endogenous *PRG4*, *SMAD3* and *SOX9*, and restored SMC markers’ expression. PRG4 modulates SMC function and osteogenic phenotype during intimal remodeling and macro-calcification in response to TGFb1 signaling, SMAD3 and SOX9 activation. The effects of PRG4 on SMC phenotype and calcification suggest its role in atherosclerotic plaque stability, warranting further investigations.

## Highlights:

Using clinical data, animal models and cell culture, our study shows that:

- Proteoglycan 4 (PRG4) induction by smooth muscle cells (SMCs) appears as an early reaction to vascular intimal remodeling, preceding, and, likely facilitating, the later formation of macro-calcifications.
- Osteogenic and inflammatory growth factors, lipids, high calcium and particularly high phosphate conditions induce PRG4 expression regulated by SMAD3 and SOX9 transcription factors, which accompanies the osteogenic phenotypic switch of SMCs.
- As a feedback loop, PRG4-enriched extracellular matrix leads to the recovery of typical SMC markers and cellular quiescence under calcifying conditions.
- The association among PRG4, SMC phenotypic modulation and atherosclerotic plaque calcification warrants further translational investigations to explore PRG4 as a clinical marker of plaque phenotype.

## 1. Introduction

Advanced and unstable atherosclerotic plaques are characterized by enhanced inflammation, enlargement of the lipid rich necrotic core (LRNC), smooth muscle cell (SMC) depletion, thinning of the fibrous cap, neovascularization and intraplaque hemorrhage [1]. Calcification is another prominent process in human atherosclerosis, where complete knowledge of its clinical significance as well as underlying molecular mechanisms is lacking. While micro-calcification has been linked to unstable lesions in patients [2] and plaque inflammation in ApoE^−/−^ mice [3], it has not yet been conclusively shown how macro-calcification impacts plaque stability. Whereas quantification of plaque calcification in coronary arteries (CAC scoring) is a surrogate risk marker for cardiovascular disease (CVD), we and others have shown that carotid plaque macro-calcification is intriguingly associated with a more stable molecular plaque phenotype [4,5,6,7]. Thus, a deeper investigation of the molecular mechanisms involved in vascular calcification would contribute to a better understanding of calcification in a clinical context.

We previously found enrichment of genes linked with calcification in advanced carotid plaques from asymptomatic patients and symptomatic patients on statin therapy [6,8]. Assessment of plaque calcification by computed tomography (CT) combined with analyses of plaque global gene expression revealed that macro-calcification was associated with molecular signatures typically linked to plaque stability and, in particular, genes related to a differentiated SMC phenotype, extracellular matrix (ECM) remodeling and repression of inflammation [4]. Other recent studies in mice have shown that SMCs display broad phenotypic plasticity in response to factors present in the atherosclerotic milieu and can undergo various forms of transdifferentiation, including an osteo-chondrogenic gene expression program [9,10]. However, osteogenic transdifferentiation processes in human atherosclerosis remain poorly understood, mostly due to the lack of key molecular markers for assessing cellular identity.

Recently, we discovered that Lubricin/Proteoglycan 4 (PRG4) is one of the most upregulated genes in calcified human carotid plaques, as assessed by preoperative CT imaging and validated by histology [4]. PRG4 is a glycoprotein produced in joints by synovial fibroblasts and SOX9 positive superficial zone chondrocytes, providing surface lubrication, reducing inflammation and exhibiting cytoprotective functions [11,12,13,14]. It has been shown to support endochondral bone formation in vivo [15], a process typically co-controlled by the master transcription factors SOX9 and RUNX2. Therapeutically, recombinant PRG4 has been coupled to disease-modifying effects in pre-clinical osteoarthritis models [12,16]. In plaques, we reported that *PRG4* gene expression correlated to bone metabolism and inhibition of inflammatory pathways, and the protein localized to calcified regions with activated macrophages and SMC-like cells. This finding was expanded in a cohort of patients with aortic valve stenosis, where we showed that PRG4 induction related to increased valvular fibrosis as well as valve interstitial cell calcification [17]. In addition, the osteogenic factor TGFb1 stimulated *PRG4* expression in valve interstitial cells, as previously shown for articular cartilage [18].

Here, we hypothesized that *PRG4* expression marks a cytoprotective SMC response to the atherosclerotic milieu and is involved in the formation of macro-calcifications. Our hypothesis was evaluated in two independent human biobanks comprising the full spectrum of atherosclerotic disease stages. Functionally, we investigated the role of PRG4 by in vivo studies of intimal hyperplasia in response to vascular injury in rats and mice, as well as studies of calcification in mouse atherosclerosis. Mechanistically, PRG4 was studied in the context of SMC phenotypic modulation and calcification in vitro, including PRG4 silencing and addition of recombinant human PRG4 protein. The results of this study demonstrate that PRG4 is an important factor in SMCs undergoing an osteogenic phenotypic switch during early vascular remodeling, preceding atherosclerotic plaque macro-calcification.

## 2. Materials and Methods

The data that support the findings of this study are available from the corresponding author upon reasonable request.

### 2.1. Human Material

Patients undergoing surgery for carotid artery stenosis (>50% NASCET, The North American Symptomatic Carotid Endarterectomy Trial) [19] at the Department of Vascular Surgery, Karolinska University Hospital, Stockholm, Sweden were consecutively enrolled in the study and clinical data recorded on admission. Symptoms were defined as transitory ischemic attack, minor stroke and amaurosis fugax. Patients without qualifying symptoms within 3 months prior to surgery were categorized as asymptomatic and an indication for carotid endarterectomy based on results from the Asymptomatic Carotid Surgery Trial (ACST) [20]. Carotid endarterectomies (carotid plaques) were collected at surgery and retained within the Biobank of Karolinska Endarterectomies (BiKE). The study cohort demographics, details of sample collection and processing and transcriptomic analyses by microarrays were as previously described in detail [6,21,22]. Of note, only 12.5% of the patients included in this analysis were treated with anticoagulants and 7.5% were on warfarin, specifically. Briefly, plaques were divided transversally at the most stenotic part—the proximal half of the lesion used for RNA preparation, while the distal half was processed for histology. Gene expression analyses were performed in two batches using Affymetrix HG-U133 plus 2.0 Genechip arrays (Santa Clara, CA, USA) and Affymetrix HG-U133a Genechip arrays (Santa Clara, CA, USA). Gene expression data were recorded on a log2 scale following robust multi-array average normalization and probe set-filtering based on signal intensity and batch effect correction. The full data set is available from Gene Expression Omnibus (accession number GSE21545). All human samples were collected with informed consent from patients; studies were approved by the Ethical Review Board and follow the guidelines of the Declaration of Helsinki.

The SOKRATES study comprises progressive aortic atherosclerotic lesions collected during organ transplantation, covering all age groups and the whole spectrum of atherosclerotic disease. Briefly, two centimeters of excessive aorta proximal and distal from the ostium of the renal artery was removed and lesions were classified according to modified American Heart Association (AHA) classification [23] as proposed by Virmani et al. [24]. Details of sample collection, demographics of the cohort along with tissue processing and full histological classification have been described previously [25]. SOKRATES study is performed in accordance with the guidelines of the Medical and Ethical Committee in Leiden, The Netherlands and the code of conduct of the Dutch Federation of Biomedical Scientific Societies.

### 2.2. Animal Studies

The simple randomization method was applied in all animal studies and group results were analyzed in a blinded fashion. Only male animals were used to ensure better control over the possible variability of data related to sex. There was no exclusion of individual animals from the study and analyses were conducted for all available samples in each experiment. All animal care and experimental procedures were performed in accordance with the guidelines for use of experimental animals and were approved by the local animal experimentation ethics committee.

#### 2.2.1. Mouse Carotid Ligation Model

Twelve-week-old male Apoe^−/−^ knockout mice were used for partial ligation of the right carotid artery as previously reported [26]. In brief, we inflicted an incomplete ligation (Vicryl 5-0 suture; Ethicon Endo-Surgery) of the common right carotid artery (proximal to bifurcation) for 4 weeks, triggering intimal hyperplasia and stable carotid atherosclerotic lesion development. The animals were anesthetized using isoflurane/O_2_ (2:1). Subcutaneous injection of buprenorphine (0.1 mg/kg) was applied before and after surgery for pain relief. Upon sacrifice, carotid arteries were stored in RNAlater (Invitrogen, Carlsbad, CA, USA) at −20 °C for RNA extraction or embedded in OCT compound and fresh-frozen for immunohistochemistry. The RNA mouse transcriptome array (MTA; Affymetrix 902514) was performed according to the manufacturer’s instructions, followed by differential expression analysis using Affymetrix transcriptome analysis console version 3.0. In this study, the dataset was queried for values of a select number of transcripts. Results are given as relative mRNA expression in a.u. log2-transformed fold changes, compared with background intensity.

#### 2.2.2. Rat Carotid Artery Balloon Injury

Male Sprague–Dawley rats (*n* = 69, purchased from Charles River, Scanbur Research A/S, Sollentuna, Sweden) were subjected to carotid artery balloon injury as previously described [27,28]. In brief, the left carotid artery was dissected under isoflurane inhalation anesthesia, an arteriotomy performed in the external carotid artery and the common carotid artery de-endothelialized 3 times with a 2F Fogarty catheter. Animals were euthanized with isoflurane at various time points. Both the left (injured) and contralateral, right (uninjured) common carotid arteries were harvested. Arteries were rinsed with PBS to remove blood. Eight additional animals were sacrificed and uninjured carotid arteries used as controls (intact). Analgesics (Buprenorphine, 0.01 mg/kg, Temgesic^®^, RB Pharmaceuticals Ltd., Berkshire, UK) were administered when needed. Upon sacrifice, both injured and uninjured arteries were harvested for transcriptomic and histological analyses. Total RNA of appropriate quality, purity and integrity (RIN 7–10, A260/280 1.7–2.0, A260/230 0.7–1.5) was used for microarray transcript profiling with Affymetrix GeneTitan Rat Gene ST v1.1 arrays.

#### 2.2.3. Mouse Atherosclerotic Calcification Model

Male C57BL6/J ApoE^−/−^ mice (*n* = 66) provided by Maastricht University were used in this study. Animals were 8 weeks of age when entering the study and housed in standard cages with free access to water and food. Atherosclerosis was induced as previously described [29] using a standard vitamin K-deficient Western type diet (WTD; 0.25% cholesterol, 15% cocoa butter and 1% corn oil; AB diets (4021.40), Woerden, The Netherlands). The control group additionally received vitamin K1 (100 mg/g) while the warfarin group received warfarin (3.0 mg/g) + vitamin K1 (1.5 mg/g), to avoid warfarin effects on the liver and prevent bleeding while introducing vitamin K-deficiency in the vasculature. Mice were sacrificed after 7, 13 or 19 weeks to perform immunohistochemical analysis.

### 2.3. In Vitro Assays

For cytokine stimulation, silencing, calcification, migration and proliferation assays primary human carotid smooth muscle cells (HCtSMCs) were sourced from Sigma-Aldrich (#3514-05A, Cell Applications, San Diego, CA, USA) and primary human aortic smooth muscle cells (HAoSMCs) were obtained from Lonza (#CC-2571, LOT NO. 0000369150, ascending aorta, Basel, Switzerland). In addition, HAoSMCs were isolated at Maastricht University from aortic wall biopsies classified as normal tissues, obtained during aneurysm surgeries [30,31]. Briefly, intima, fat and connective tissue was carefully removed before cutting the sample into small fragments and placing into laminin (#L2020, Sigma-Aldrich, St. Louis, MO, USA) coated plates. The pieces were cultured in M199 medium containing 20% FBS, 1% PS and 1% Amphotericin B (#15290-026, Gibco, Waltham, MA, USA). When outgrowing cells reached confluency, they were passaged to laminin coated T25 flasks, tested for mycoplasma and characterized by immunohistochemistry for expression of SMC markers (CNN1, SM22a, SMA, p-MLC). All cells were used at passages 7–9.

#### 2.3.1. Cytokine Stimulations

Cytokine stimulation was conducted as previously described [22]. In brief, commercial HCtSMCs or HAoSMCs (200,000 cells per well) from one donor were plated on 6-well plates and left to adhere. After overnight serum–starvation, cells were separately treated with a panel of cytokines and growth factors (PDGFB, #PHG0044, Gibco, 50 ng/mL; TGFβ1, #T7039, Sigma, 20 ng/mL; TNFα, PeproTech, Rocky Hill, NJ, USA, 20 ng/mL; IFNγ, #285-IF-100, R&D Systems, 20 ng/mL; IGF1, #I3769, Sigma, 20 ng/mL; IGF2, #I2526, Sigma, 20 ng/mL) and collected at several time-points (2, 4, 8 and 24 h) for RNA isolation and qPCR analyses.

#### 2.3.2. Transfection and PRG4 Silencing

Commercial HAoSMCs from one donor were plated in 6-well plates at sub-confluence (200,000 cells per well) and left to adhere overnight. Upon induction of osteogenic transformation by 20 ng/mL TGFβ1 (#T7039, Sigma) stimulation for 24 h in OptiMEM medium (#51985-026, Life Technologies, Carlsbad, CA, USA), gene silencing was achieved via treatment with 50 nM siRNA (PRG4 #s19926, SOX9 #s532695, SMAD3 #s8401 or scramble control #4390844, ThermoFisher, Waltham, MA, USA) per well. SiRNA transfection was conducted by mixing with Lipofectamin (#15338100, ThermoFisher) and OptiMEM, allowing droplets to form for 30 min at room temperature. After 48 h, cells were harvested for RNA extraction.

#### 2.3.3. Migration Assay

To assess SMC migration, an in vitro scratch assay was conducted as previously described [32]. In short, a straight scratch was created in a monolayer of commercial HAoSMCs from one donor growing in 6-well plates using a 1000 μL pipette tip. Thereafter, 100 μg/mL rhPRG4 was added to the basal growth medium (5% FBS, #CC-3181, Lonza). Migration was continuously monitored, and images were taken after 0, 8, 18 and 24 h. Wound closure was quantified by measuring the distance between the migration fronts at 3 random locations of 3 wells per time point and condition.

#### 2.3.4. Proliferation Assay

Commercial HAoSMCs from one donor were plated in 96-well plates (6000 cells per well) and left to adhere overnight. After 6 h of serum starvation, cells were incubated in basal growth medium (5% FBS, #CC-3181, Lonza, Basel, Switzerland) with or without addition of 50 ng/mL PDGFB (#PHG0044, Gibco, Waltham, MA, USA), 20 ng/mL TGFβ1 (#T7039, Sigma-Aldrich, St. Louis, MO, USA) or 100 μg/mL rhPRG4. Cell proliferation was assessed in a microplate reader via a colorimetric immunoassay based on BrdU incorporation during DNA synthesis (#11647229001, Roche, Basel, Switzerland) after 4, 8, 16 and 24 h, according to the manufacturer’s protocol.

#### 2.3.5. Calcification Assay

Commercial HAoSMCs from one donor as well as HAoSMCs isolated from three thoracic aneurism patients were seeded in 6-well plates (80,000 cells per well) and incubated in DMEM GlutaMAX (31966-021; #12077549 ThermoFisher, Waltham, MA, USA) supplemented with 3.6 mM Ca and 2.5% FBS or 2.6 mM PO_4_ and 5% FBS, for 12 days. Medium was refreshed after 3 days. Respective controls contained 2.5% or 5% FBS and MilliQ water. For longitudinal quantification of calcification formation, a probe containing Fetuin-A coupled with Alexa-fluor 546 (kindly provided by W. Jahnen-Dechent, RWTH Aachen, Aachen, Germany) was used and nuclei stained by Hoechst. Sequential imaging was performed on a Cytation 3 System (BioSPX, Abcoude, The Netherlands). Cells were harvested after 5 and 9 or 12 days for RNA extraction.

#### 2.3.6. OxLDL Assay

Lipid-loading assays were performed in commercial HAoSMCs from one donor according to a previously published protocol [33]. Briefly, cells were stimulated with copper oxidized LDL (20 μg/mL) in OptiMEM medium (#51985-026, Life Technologies, Carlsbad, CA, USA, 1% FBS) for 6, 24, 48 and 72 h. Cells incubated with 1% FBS without oxLDL treatment served as controls.

### 2.4. Recombinant Human PRG4

Full length recombinant human PRG4 (rhPRG4) was provided by Lubris BioPharma [34,35]. Briefly, rhPRG4 protein was derived from Chinese Hamster Ovary cells (Lubris BioPharma, LLC, Framingham, MA, USA) [34]. The gene encoding the full length 1404 amino acid human PRG4 was inserted into plasmid vectors, commercially available at Selexis SA (Geneva, Switzerland). Subsequently, the conditioned media was subjected to ultrafiltration/diafiltration and a 3-step chromatographic purification process [14]. In this study, rhPRG4 was added to the culture medium after cells had attached over night at a concentration of 100 μg/mL (stock solution 1 mg/mL in PBS). For calcification assays, rhPRG4 was added 24 h prior to calcifying conditions.

### 2.5. RNA Extraction and Gene Expression Analyses by Quantitative PCR (qPCR)

RNA was prepared either from tissues using Qiazol Lysis Reagent (Qiagen, Hilden, Germany) or from cells using RLT (#79216, Qiagen, Venlo, The Netherlands) buffer containing 1% 2-Mercaptoethanol (M3148, Sigma) and purified by the RNeasy Mini kit (#74106, Qiagen, Venlo, The Netherlands), including DNase digestion. The concentration was measured using Nanodrop ND-2000 (Thermo Scientific, Waltham, MA, USA) and quality estimated by a Bioanalyzer capillary electrophoresis system (Agilent Technologies, Santa Clara, CA, USA). For qPCR, total RNA was reverse-transcribed using High Capacity RNA-to-cDNA kit (#4387406, Applied Biosystems, Carlsbad, CA, USA). PCR amplification was performed in 96-well or 384-well plates in a 7900 HT real-time PCR system (Applied Biosystems, Carlsbad, CA, USA), using TaqMan^®^ Universal PCR Master Mix (#4324018, Applied Biosystems, Carlsbad, CA, USA) and TaqMan^®^ Gene Expression Assays (PRG4 probe Hs00981633_m1, SOX9 probe Hs00165814_m1, SMAD3 probe Hs00969210_m1, BMP2 probe HS00154192_m1, MYOCD probe Hs00538076_m1, ACTA2 probe Hs00426835_g1, MYH11 probe Hs00975796_m1, CNN1 probe Hs00959434_m1, #4331182 Thermo Fisher, Waltham, MA, USA). All samples were measured in duplicate. Results were normalized to the equal mass of total RNA as well as the Ct values of RPLPO (Hs99999902_m1) housekeeping control. The relative amount of target gene mRNA was calculated by the 2^−ΔΔCt^ method.

### 2.6. Histological Analyses

To enable processing for histological stainings, macro-calcified plaques were de-calcified after fixation in Modified Decalcification Solution (HL24150.1000) for 4–6 days depending on plaque size. Tissues were then rinsed in distilled water, dehydrated and paraffin-embedded.

#### 2.6.1. Antibodies

The following primary antibodies were used in the study: PRG4 (HPA028523, Sigma-Aldrich, St. Louis, MO, USA), SMA (M0851, DAKO, Santa Clara, CA, USA), CD68 (M0876, DAKO, Santa Clara, CA, USA), TRAP (LS-C87845, LS BIO, Seattle, WA, USA), SOX9 (AMAB90795, Sigma-Aldrich, St. Louis, MO, USA; Ab26414, Abcam, Cambridge, UK), RUNX2 (AMAB90591, Sigma-Aldrich, St. Louis, MO, USA), VWF (M0616, DAKO, Santa Clara, CA, USA).

#### 2.6.2. Immunohistochemistry (IHC)

For staining of human plaques, IHC reagents were from Biocare Medical, Pacheco, CA, USA. Isotype rabbit and mouse IgG were used as negative controls. In brief, 5 μm sections were deparaffinized in Tissue Clear and rehydrated in ethanol. For antigen retrieval, slides were subjected to high-pressure boiling in DIVA buffer (pH 6.0) or TE buffer (pH 9.0). After blocking with Background Sniper, primary antibodies diluted in a Da Vinci Green solution were applied and incubated at room temperature for 1 h. A double-stain probe-polymer detection kit (Mach 2) containing both alkaline phosphatase and horseradish peroxidase was applied, with subsequent detection using Warp Red and Vina Green. All slides were counterstained with hematoxylin (HTX QS, H-3404, Vector Laboratories Inc., Burlingame, CA, USA), dehydrated and mounted in Pertex (Histolab, Gothenburg, Sweden). Images were taken using a Nikon Eclipse E800 microscope.

For staining of mouse sections, sequential 5 μm slides were rehydrated, antigens were retrieved by boiling in a TriSodiumCitrate buffer (pH 6.0). Primary antibodies were visualized with a Nova-RED substrate (Vector #SK-4800, Vector Laboratories Inc., Burlingame, CA, USA). Sections were counterstained with hematoxylin (#4085-9002, Klinipath, VWR, Radnor, PA, USA) and mounted with entellan (#7961, Burlington, MA, USA).

#### 2.6.3. Semi-Quantitative IHC Scoring

All slides used for quantification of staining intensities were imaged with equal settings and blinded semi-quantitative evaluation of staining intensity (content) within the intimal plaque and media was performed according to a four-grade scale: 0—no staining signal, 1—weak signal or a few cells stained, 2—medium or strong signal localized in a certain area, 3—strong staining of the whole section area.

#### 2.6.4. Immunofluorescence (IFL)

Paraffin-embedded slides were deparaffinized in Tissue Clear, rehydrated in ethanol, and thereafter permeabilized with 0.1% Triton X-100/PBS for 5 min. Blocking was done with 10% normal horse serum (NHS, #H0146, Sigma-Aldrich, St. Louis, MO, USA). Sections were then incubated with primary antibodies diluted in 10% NHS overnight at 4 °C, washed with TBS and counterstained with Alexa Fluor 488- or 568-conjugated secondary antibodies (Invitrogen, Carlsbad, CA, USA). Nuclei were stained with diamidino-2-phenylindole (DAPI) and mounted with fluorescent mounting medium (#S3023, DAKO, Santa Clara, CA, USA). Images were taken using a Nikon Eclipse E800 (Nikon Instruments Inc., Melville, NY, USA) microscope equipped with a CoolLED pE-300 lite light source.

### 2.7. Computed Tomography (CT) Angiography Image Analysis

Carotid plaques were assessed in pre-operative CT angiographies using a semi-automated, histology-validated software as previously described (The vascuCAP^®^ (Elucid Bioimaging Inc., Boston, MA, USA) software) [36,37,38,39,40,41]. In brief, reconstructed images were analyzed in a blinded fashion by one observer (EK) to characterize plaque structure and composition (plaque morphology) [4] creating 3D segmentations with improved resolution and soft tissue plaque component differentiation. A patient-specific 3D point spread function restored image intensities to represent the original tissues, which mitigates artefacts and enables discrimination of tissue types such as LRNC, for which overlapping densities were classified by expert-annotated histology. To avoid limitations of fixed thresholds, accuracy was achieved by algorithms that account for distributions of tissue constituents rather than assuming constant material density ranges. The common and internal carotid artery were defined as target, lumen and wall evaluated automatically or edited manually when needed.

Defined tissue components included: LRNC, CALC and MATX (representing plaque tissue not detected as either LRNC or CALC), quantified with their absolute volume (Vol) and ratio of the total wall volume (VolProp). Structural features included: plaque volume (Vol), plaque burden volume and area (ratio of the total vessel volume or area) and stenosis degree (NASCET).

### 2.8. Bioinformatic and Statistical Analyses

Distribution of data was assessed using the Shapiro-Wilks normality test. Comparative statistics between time-points and groups was conducted using 2-way ANOVA or simple comparison between groups by a *t*-test. Pearson and Spearman rank correlations were calculated for data of normal and non-normal distributions, respectively, to determine the association between mRNA expression levels from microarrays. Multiple linear regression analysis was performed between mRNA expression and quantitated CT parameters. All statistical analyses were performed with GraphPad Prism 9. Pearson correlations between mRNA expression levels were illustrated in correlation plots performed by use of R with additional packages installed [42,43]. Quantifications of in vitro scratch and calcification assays based on images were conducted in Fiji ImageJ.

Additional Material and Methods are described in the Supplementary Data file.

## 3. Results

### 3.1. Study Design and Correlation of PRG4 with Human Plaque Composition

**Figure 1:**
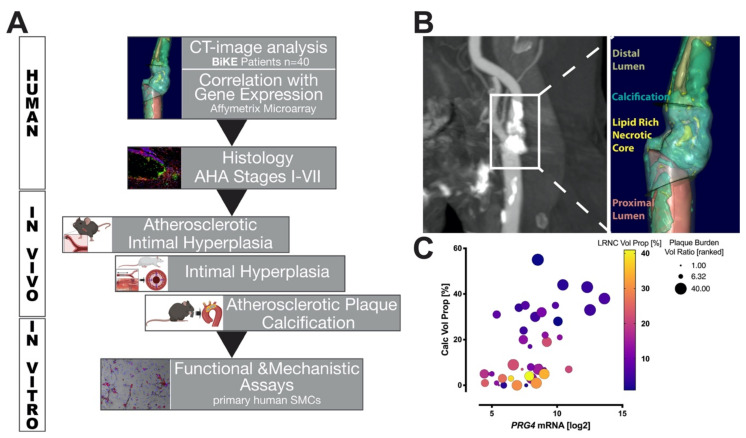
Study workflow and characterization of PRG4 expression in relation to plaque morphology. (**A**) Tissue composition of human carotid plaques was determined and plaque morphology correlated to *PRG4* gene expression. PRG4 protein was assessed in relation to human atherosclerosis progression and its role in intimal hyperplasia, atherosclerotic plaque development and plaque calcification characterized using rodent models in vivo. Ultimately, pathways of *PRG4* activation during osteogenic SMC modulation were investigated in vitro. (**B**) Illustration of quantitative plaque composition analysis using vascuCap software (Boston, MA, USA), based on pre-operative computed tomography angiography images; (**C**) *PRG4* mRNA plaque expression in relation to plaque calcification volume proportion (CALCVolProp) and lipid rich necrotic core volume proportion (LRNCVolProp) as well as plaque burden volume (PlaqueBurdenVolRatio). AHA-American Heart Association. Correlations assessed by Pearson coefficient (*n* = 40 patients).

A cohort of patients with end-stage, high- and low- calcified carotid plaques (*n* = 40), as previously described [4], was used to correlate *PRG4* mRNA expression from plaque microarrays with quantitative morphological tissue composition as assessed by image analysis of pre-operative CTA (Figure 1A). We proceeded to confirm PRG4 protein expression and investigate its localization within end-stage human carotid plaques, as well as its relation to disease progression in pathological samples of aortic atherosclerotic lesions representing AHA-stages I–VII. To assess the role of PRG4 during early plaque development, we used two ApoE^−/−^ mouse models representing atherosclerotic plaque calcification and intimal hyperplasia, as well as a longitudinal rat balloon-injury carotid artery intimal hyperplasia model. Mechanisms of *PRG4* induction in the context of SMC activation and osteogenic modulation were investigated using human primary SMC cultures.

Quantitative plaque tissue modeling performed on CTA images, utilizing the vascuCap software (Figure 1B), showed a significantly positive independent correlation between *PRG4* gene expression and calcification volume proportion (CALC Vol Prop: calcified volume as a proportion of total wall volume, r = 0.561, *p* < 0.001). *PRG4* expression was also positively corelated with plaque burden volume ratio (wall volume divided by vessel volume inclusive of lumen and wall, r = 0.452, *p* = 0.003) and showed a negative trend with lipid rich necrotic core volume (LRNC Vol Prop: LRNC volume as a proportion of total wall volume, r = −0.268, *p* = 0.095), but these associations were not independent (Figure 1C and Table S1).

### 3.2. PRG4 Is Detectable in Human Adaptive Intimal Thickening and Intimal Xanthomas

**Figure 2:**
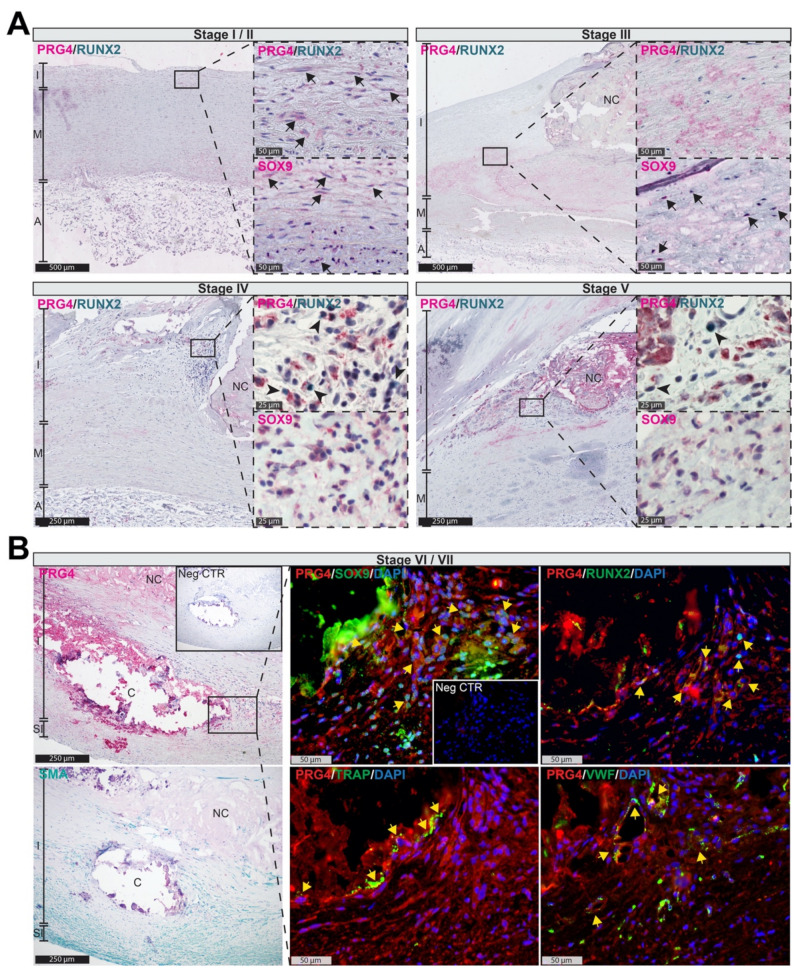
PRG4 is upregulated during human atheroprogression and implicated in osteo-chondrogenic plaque remodeling. (**A**) Immunohistochemistry on sections representing AHA stages I to V of human atherosclerotic pathology. PRG4 signal was detected in stages I/II as intracellular (arrows) and III as extracellular, in areas with SOX9^+^ cells (arrows). In stages IV and V, PRG4^+^ areas also overlapped with RUNX2^+^ cells (arrowheads). Hematoxylin was used as counterstain; (**B**) Immunofluorescence on late-stage (AHA grade VI and VII) plaques. PRG4 signal overlaid (yellow arrows) with SOX9, RUNX2 as well as TRAP and VWF. Images show 10× magnification, enlarged images 20×/40×. Insets show corresponding isotype negative control. AHA—American Heart Association; A—adventitia; C—calcification; I—intima; M—media; NC—necrotic core; SI—subintima. (**A**) Images representative of *n* = 2 patients per AHA-stage, (**B**) images representative of *n* = 5 patients.

To confirm PRG4 protein expression and characterize its localization during lesion formation, we performed in situ assessment of sections representing human atheroprogression throughout AHA stages I to VII [44,45]. The PRG4 signal was already detectable in stages I/II (intracellular) and III (extracellular) localizing in the same areas as SOX9 on consecutive sections, (Figure 2A, arrows) as well as tissue regions that we previously identified as SMC rich [22]. During stages IV and V, PRG4^+^ areas overlapped with RUNX2^+^ cells (Figure 2A, arrowheads) in the shoulder regions of calcifying lesions. Finally, in plaques of end-stage atherosclerotic patients (AHA stage VI and VII), PRG4 signal overlayed with SOX9, RUNX2, TRAP and VWF expression within SMA^+^ areas and neovessels surrounding the macro-calcifications as highlighted by immunofluorescent staining (Figure 2B). These findings suggest that *PRG4* upregulation is implicated in early osteogenic intimal remodeling preceding the formation of macro-calcifications in human atheroprogression.

### 3.3. PRG4 Is Expressed Early during Vascular Remodeling In Vivo

**Figure 3:**
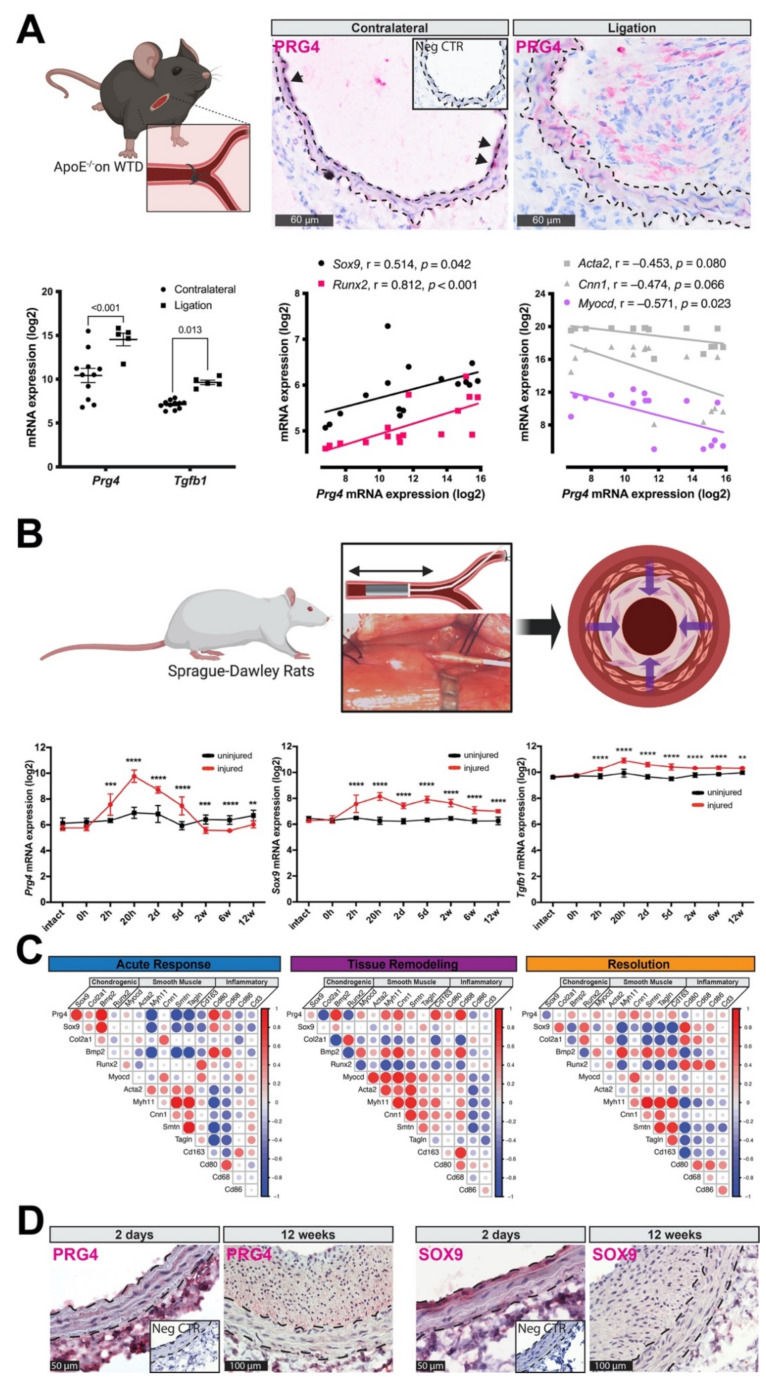
PRG4 is implicated during early intimal vascular remodeling in vivo. (**A**) IHC detected a sporadic presence of PRG4 protein in carotid arteries of ApoE^−/−^ mice. However, in partially ligated carotids, there was an increase of signal throughout the media (dashed lines) and neo-intima. Hematoxylin was used as counterstain. *Prg4* and *Tgfb1* mRNA expression was significantly increased after ligation compared to contralateral controls. *Sox9* and *Runx2* mRNA levels were positively correlated with *Prg4* expression, while typical markers for SMC quiescence showed a negative correlation (grey color indicating correlations not reaching statistical significance). (**B**) In rat carotid balloon injury, *Prg4* mRNA was significantly elevated together with *Sox9* expression. Its levels peaked at 20 h and gradually normalized thereafter. This model also showed an upregulation of *Tgfb1* at two hours and throughout the healing process. (**C**) Correlograms of *Prg4* with expression of major markers (positive correlation—red, negative correlation—blue, circle size—Pearson coefficient) indicate: strong positive correlation of *Prg4* with osteogenic genes (i.e., *Sox9, Bmp2*), and inflammatory markers (i.e., *Cd80*, *Cd68*), but negative correlation with sensitive markers of contractile SMCs (*Myh11, Smtn, Tagln*) during early acute response to injury (0–2 h). During the tissue remodeling phase (20 h–5 d), decreasing *Prg4* expression positively correlated with *Bmp2,* as well as typical contractile and inflammatory markers. However, it negatively correlated with the osteogenic transcription factor *Runx2*. Concomitantly with the resolution of injury response (2–12 weeks), *Prg4* expression levels were downregulated and showed positive trends in association with recovery of typical SMC markers. (**D**) Immunohistochemistry showed that the presence of PRG4 protein preceded intimal remodeling already at two days post injury, but persisted within the ECM even after 12 weeks. The SOX9 signal was strong within the luminal medial layer after injury and not any more detectable on protein level 12 weeks after injury. Images show 40× magnification. Insets show corresponding isotype negative control. h—hours, d—days, w—weeks. Intact arteries used as controls. Plots show (**A**) mean with SEM or correlation, respectively; (**B**) mean with SD. Statistical difference between treatment-groups assessed by *t*-test; correlation assessed by (**A**) Spearman coefficient (*n* = 16 mice), (**B**) Pearson coefficient (*n* = 69 rats). ** *p* < 0.01, *** *p* < 0.001, **** *p* < 0.0001.

Considering that SMCs are the major cell type responsible for intimal remodeling and undergo various phenotypic transformations in this process [22,46], we investigated the expression of *Prg4* in two established models of SMC modulation and hyperplasia, typically not associated with calcification: (i) during intimal hyperplasia formation in ApoE^−/−^ mice undergoing partial carotid ligation, as well as (ii) in a longitudinal rat carotid balloon injury model. Intimal hyperplasia in ApoE^−/−^ mice was associated with a significant increase of *Prg4* and *Tgfb1* mRNA expression four weeks after surgery, compared to contralateral controls (Figure 3A). *Sox9* and *Runx2* mRNA levels were not significantly increased at this time point but positively correlated with *Prg4* expression (*Sox9* r = 0.514, *p* = 0.042; *Runx2* r = 0.812, *p* < 0.001), while typical markers for contractile SMCs showed negative correlation (*Myocd* r = −0.517, *p* = 0.023; *Acta2* r = −0.453, *p* = 0.080; *Cnn1* r = −0.474, *p* = 0.066). There was a strong PRG4 protein signal in the media and neo-intima of partially ligated carotids, while few cells within the media of contralateral controls were positively stained.

Transcriptomic analysis of rat carotid arteries after balloon injury revealed that *Prg4* mRNA together with *Sox9* and *Tgfb1*, was already significantly upregulated two hours after injury compared to uninjured artery, peaked at 20 h, and remained elevated up to five days (Figure 3B). The expression of these genes thereafter gradually declined and returned close to baseline levels at 12 weeks. Moreover, we found a strong positive correlation between *Prg4* mRNA expression, chondrogenic- (*Sox9, Bmp2*) and macrophage-markers (*Cd68*), but a negative correlation to more sensitive markers of contractile SMCs (*Myh11, Smtn, Tagln*) during the acute response to injury (0–2 h; Figure 3C). During the tissue remodeling phase (20 h to 5 d), *Prg4* expression positively correlated with *Bmp2*, typical SMC and inflammatory markers. However, *Prg4* expression negatively correlated with the osteogenic transcription factor *Runx2*. Concomitantly with the resolution phase of the injury response (2–12 weeks), *Prg4* levels returned to a baseline and showed a positive trend in association with the recovery of typical SMC markers. Immunohistochemistry of injured arteries confirmed PRG4 protein expression early after injury and showed staining within the media preceding intimal remodeling, which persisted in the intima even after 12 weeks (Figure 3D). The SOX9 signal was strong within the luminal medial layer early after injury and decreased at later time points.

### 3.4. Accumulation of PRG4 Precedes Intimal Macro-Calcification In Vivo

**Figure 4:**
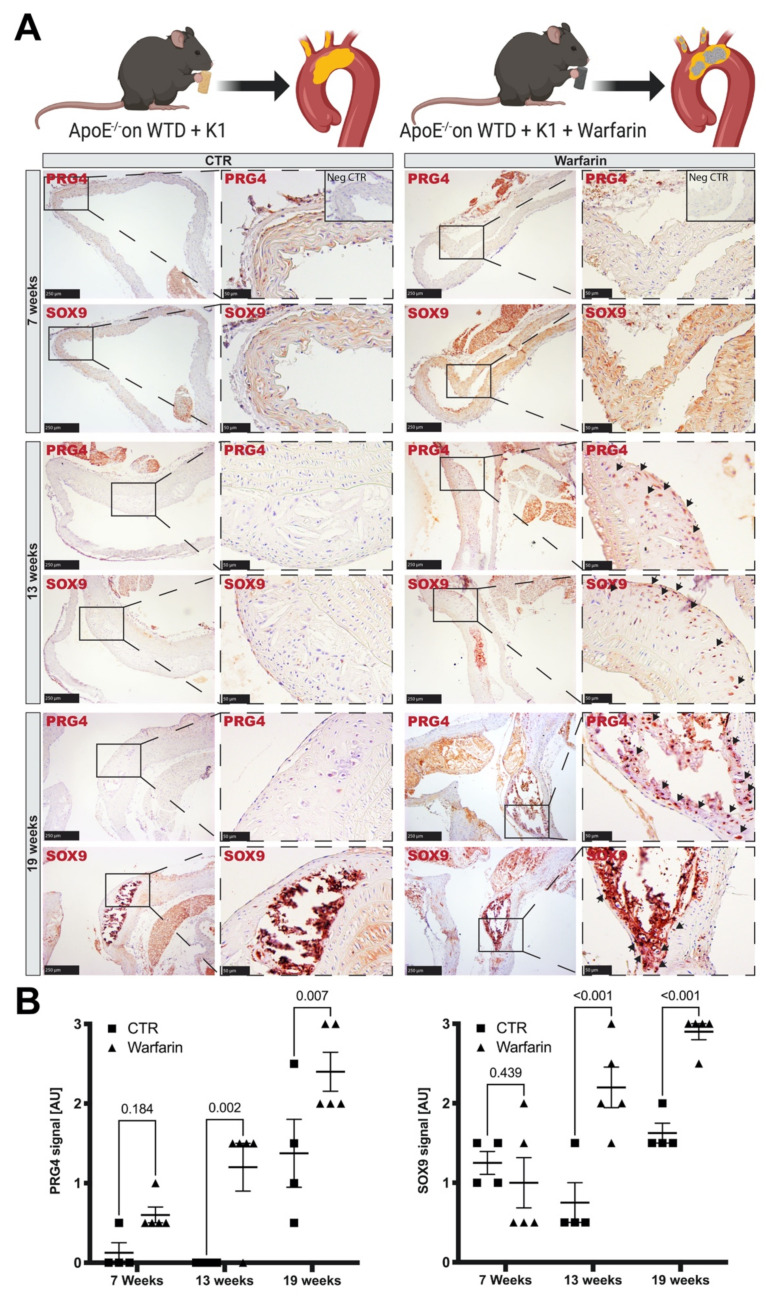
PRG4 enrichment precedes the development of atherosclerotic plaque macro-calcifications in vivo. (**A**) ApoE^−/−^ mice receiving a Western type diet supplemented with warfarin and vitamin K1 developed atherosclerotic plaques with nodular calcifications over the course of 19 weeks. While there was no significant increase in PRG4 and SOX9 signal at seven weeks, PRG4^+^ and SOX9^+^ cells were abundant in plaques after 13 weeks compared to control animals (arrows). PRG4 and SOX9 staining preceded the development of severe calcification at 19 weeks but were even more prominent within these areas at this late time-point. (**B**) Semi-quantitative scoring of IHC signal on sections from CTR (*n* = 4 per time point) and warfarin treated mice (*n* = 5 per time point). Images show 10× magnification, enlarged areas 20×. Insets show corresponding isotype negative controls. Plots show mean with SEM. A statistical difference assessed by 2-way ANOVA. CTR-ApoE^−/−^ mice on a Western type diet supplemented with vitamin K1.

End-stage human atherosclerotic plaques are characterized by intimal calcification and often contain macro-calcified nodules [23,25,47]. To investigate the role of PRG4 during atheroprogression and development of intimal macro-calcifications in vivo, we utilized a previously characterized model [29] where ApoE^−/−^ mice, receiving a Western type diet supplemented with warfarin, develop severe calcifications in the aortic arch and brachiocephalic trunc. Histological analysis of these areas (Figure 4A) showed abundant PRG4 and SOX9 positive cells after 13 weeks. At 19 weeks, these cells were detected within and surrounding highly calcified regions. In some cases, PRG4 staining was preceded by widespread SOX9 signal throughout the whole vessel wall of warfarin treated animals already after seven weeks. Plaques of control animals showed a significantly lower signal for PRG4 and SOX9 during the later stages of plaque development, as assessed by semi-quantitative IHC scoring (Figure 4B).

Taken together, our data from murine studies illustrate the early enrichment and continuous role of PRG4 via osteogenic expression patterns in SMCs during the process of intimal remodeling, towards macro-calcification typical for late-stage atherosclerosis. This suggests that pathways related to TGFB1 and SOX9 could be involved in PRG4 associated osteogenic regulation.

### 3.5. TGFb1, SMAD3, and SOX9 Control PRG4 Induction in SMCs

**Figure 5:**
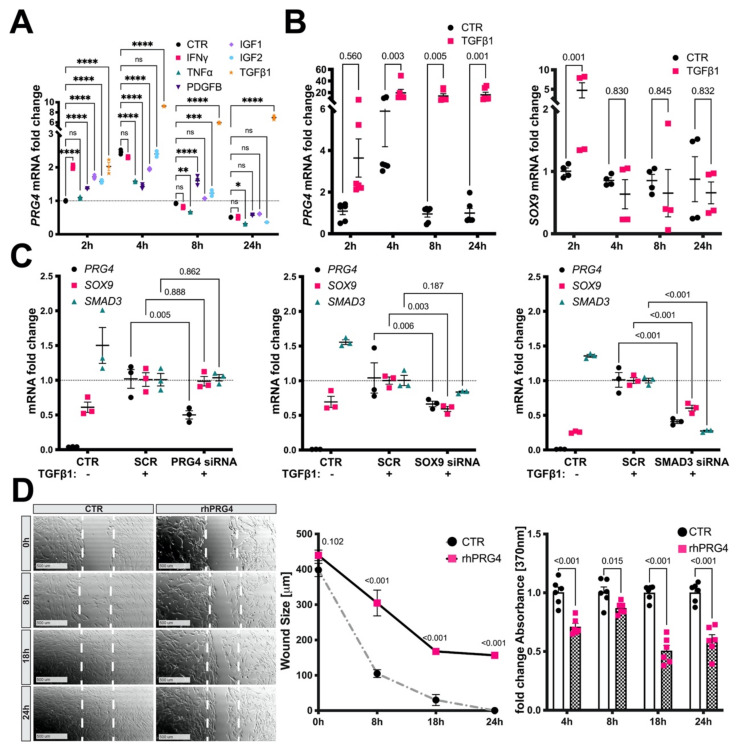
Endogenous *PRG4* expression is induced by TGFβ1 and controlled by *SMAD3* and *SOX9*, while extracellular PRG4 inhibits SMC migration and proliferation in vitro. (**A**) In HCtSMCs, stimulation by IFNγ, PDGFB, IGF1, IGF2 and TGFβ1 caused a significant early induction of *PRG4* mRNA expression, while TNFα showed no effect. (**B**) This effect was replicated in HAoSMCs, concomitantly with an upregulation of *SOX9* at 2h which rapidly returned to baseline thereafter. (**C**) While siRNA silencing of *PRG4* in HAoSMCs upon TGFβ1 treatment affected neither *SOX9* nor *SMAD3* expression, *PRG4* mRNA levels were downregulated by the knock-down of both *SOX9* and *SMAD3*. *SOX9* siRNA decreased only *PRG4* levels, while *SMAD3* siRNA decreased both *SOX9* and *PRG4*, suggesting SMAD3 to be highest upstream regulator among these genes. (**D**) HAoSMCs treated with full length rhPRG4 exhibited significantly impaired migratory capacity in the scratch assay (left and middle panel) and decreased proliferation (right panel). CTR—untreated cells in identical medium and FBS conditions; SCR—scrambled control following TGFβ1; D—day; HCtSMCs-human carotid smooth muscle cells; HAoSMCs—human aortic smooth muscle cells; rhPRG4—recombinant human PRG4. Plots show mean with SEM. Statistical difference assessed by 2-way ANOVA; (**A**) *n* = 3, (**B**) *n* = 4, (**C**) *n* = 3, (**D**) *n* = 6 experimental replicates. ns *p* > 0.05, * *p* < 0.05, ** *p* < 0.01, *** *p* < 0.001, **** *p* < 0.0001.

Based on the early enrichment of PRG4 in intimal hyperplasia and atherosclerotic lesion formation, we next explored which cytokines implicated in atherogenic and osteogenic transformation could induce *PRG4* expression in SMCs in vitro. Experiments on primary human carotid SMCs (HCtSMCs) showed a significant early induction of *PRG4* mRNA expression by IFNg, PDGFB, IGF1, IGF2 and TGFb1, while TNFa stimulation showed no effect (Figure 5A). However, TGFb1 treatment resulted in considerably higher *PRG4* levels compared to all other stimuli. The effect of TGFb1 on *PRG4* was conserved in primary human aortic SMCs (HAoSMCs), accompanied by a transient upregulation of *SOX9* at 2 h, which rapidly returned to baseline (Figure 5B). Of note, *PRG4* mRNA levels in unstimulated SMCs were mostly undetectable.

This led us to assess the impact of TGFb1 downstream signaling and SOX9 control on *PRG4* expression. We conducted siRNA knockdown experiments of SMAD3, known as an important regulator of TGFb mediated transcription in articular cartilage [48], as well as SOX9 and PRG4 following TGFb1 stimulation. While silencing of *PRG4* in HAoSMCs did not affect either *SOX9* or *SMAD3* expression, *PRG4* mRNA levels were affected by knock-down of both *SOX9* and *SMAD3,* suggesting that these transcription factors regulate PRG4 expression upon TGFb1 stimulation. *SMAD3* siRNA also decreased *SOX9* levels, suggesting SMAD3 to be upstream of SOX9 (Figure 5C). Furthermore, PRG4 siRNA had a non-significant negative effect on *ACTA2* and *CNN1* mRNA levels under these conditions (Figure S1A).

These results show that SMCs express PRG4 in vitro in response to various cytokines, but also indicate that TGFb1 signaling through SMAD3 and SOX9 is a major pathway for *PRG4* induction in SMCs.

### 3.6. Exogenous PRG4 Inhibits SMC Migration and Proliferation In Vitro

In order to characterize the functional effects of extracellular PRG4 on SMCs, exogenous rhPRG4 was added to HAoSMCs in vitro and the effects evaluated in wound healing and proliferation assays. We found a significant inhibition of SMC migration and proliferation by rhPRG4 upon stimulation with FBS (Figure 5D), TGFb1 and PDGFB (Figure S1B), showing that exogenous PRG4 has the capacity to inhibit SMC activation.

### 3.7. Calcifying SMCs Upregulate PRG4 Expression

**Figure 6:**
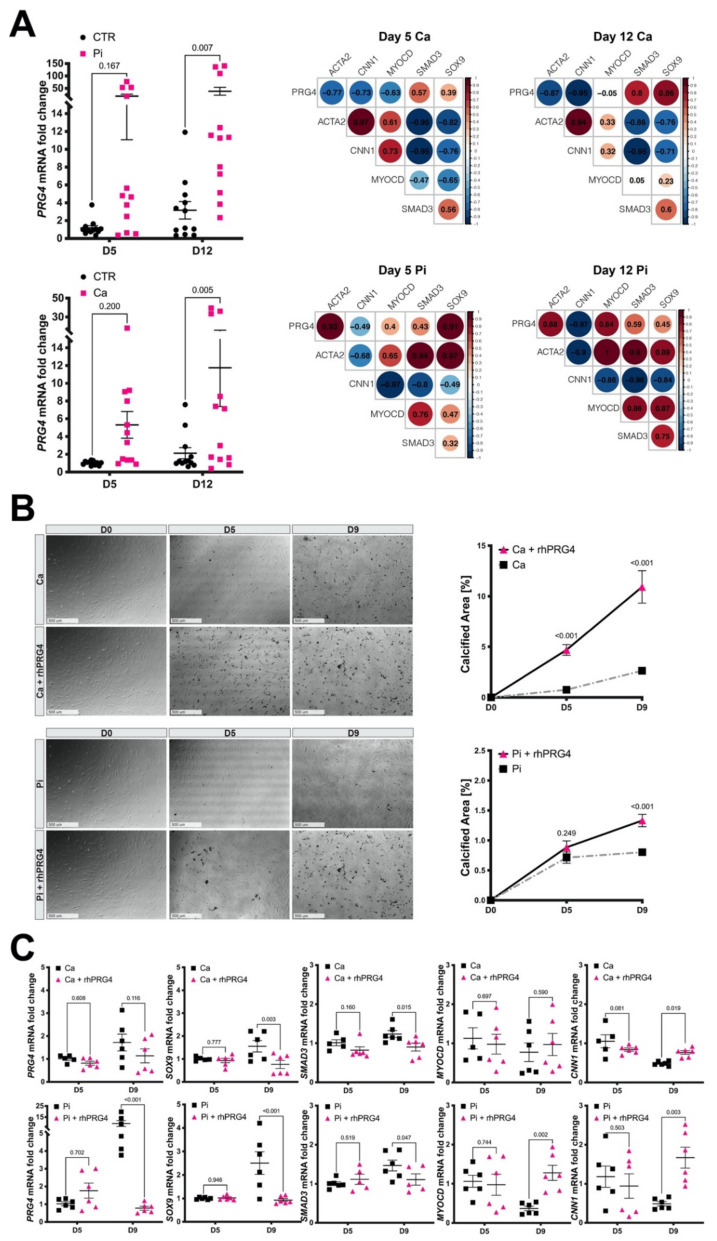
*PRG4* is induced by calcific conditions in vitro, while extracellular PRG4 protein elevates calcification but counteracts SMCs osteogenic phenotypic switch. (**A**) Treatment of HAoSMCs with high levels of calcium over the course of 12 days (Ca, 3.6 mM) resulted in upregulation of *PRG4* mRNA expression with a strong positive correlation to the expression of *SMAD3* and *SOX9*, while it negatively correlated to typical contractile markers. High phosphate conditions (Pi, 2.6 mM) resulted in even higher *PRG4* mRNA levels again positively correlating to *SMAD3* and *SOX9,* while correlations with SMC markers were partially positive (i.e., with *MYOCD* and *ACTA2*) and negative (i.e., with *CNN1*). Correlograms show: positive correlation—red, negative correlation—blue, circle size—Pearson coefficient. (**B**) Addition of rhPRG4 to the cell medium prior to calcium treatment significantly promoted the development of ectopic calcification, and the same effect was observed with high phosphate. (**C**) However, endogenous *PRG4*, *SOX9* and *SMAD3* expression was repressed after nine days, when rhPRG4 was present during calcifying treatment. RhPRG4 also prevented the downregulation of *MYOCD* and *CNN1* mRNA, in contrast to what was observed in calcifying conditions. Images show 4x magnification. CTR-untreated cells in identical medium and FBS conditions; HAoSMCs—human aortic smooth muscle cells; rhPRG4—recombinant human PRG4 protein. Plots show mean with SEM. Statistical difference assessed by 2-way ANOVA; correlation assessed by Pearson coefficient; (**A**) *n* = 3 replicates with cells from three patients and commercial cells from Lonza, (**B**,**C**) *n* = 3 replicates in primary patient cells and commercial cells from Lonza.

As PRG4 was associated with calcification and an osteogenic transition of SMCs, we next investigated the direct impact of pro-calcific conditions on *PRG4* expression in SMCs. Stimulation of HAoSMCs with either 3.6 mM Ca or 2.6 mM PO_4_ induced ectopic calcification as assessed by fetuin-A staining (Figure S2A–C) and increased *SOX9* and *SMAD3* mRNA (Figure S2B–D). Expression of typical SMC contractility markers (ACTA2, CNN1) decreased, while MYOCD remained relatively stable and *PRG4* expression was significantly upregulated, which positively correlated to *SOX9* and *SMAD3* expression. However, while the extent of calcification was higher with calcium exposure, stimulation with phosphate was able to induce higher expression levels of *PRG4* mRNA after 12 days (Figure 6A). In addition, under calcium stimulation, *PRG4* negatively correlated to *ACTA2, CNN1* and *MYOCD,* whereas, under high phosphate, *ACTA2* and *MYOCD* correlations turned positive. Of note, oxLDL loading (20 μg/mL) of HAoSMCs significantly induced *PRG4,* especially after 48 h when *SOX9* levels were increased too, showing that elevated lipid levels also have an effect on *PRG4* (Figure S3).

These data confirmed the induction of *PRG4* during osteogenic changes of SMCs under calcifying conditions, with high lipid levels likely being a contributing stimulus. Furthermore, some differences in the process induced by high levels of calcium or phosphate were seen, suggesting that high phosphate is a more potent stimulus for PRG4 expression in SMCs.

### 3.8. Exogenous PRG4 Elevates Calcification and Counteracts SMC Phenotypic Switch

Since *PRG4* was upregulated in association with calcification in vitro, we also tested the effects of exogenous PRG4 on the development of calcification nodules and SMC phenotype. Addition of rhPRG4 to the medium 24 h before treatment with calcium or phosphate significantly increased calcification (Figure 6B). No significant formation of passive precipitation could be detected in control experiments without cells (Supplementary Figure S4). Strikingly, rhPRG4-supplementation decreased endogenous *PRG4* accompanied by reduced *SOX9* and *SMAD3* expression in calcifying SMCs after nine days of both calcium and phosphate exposure. Further repression of *MYOCD* and *CNN1* was prevented and the expression of these markers restored in calcifying SMCs (Figure 6C). A similar stimulating effect on SMC contractile markers could be observed by rhPRG4 treatment only (Supplementary Figure S5).

Collectively, we show that addition of rhPRG4 promotes the recovery of SMC contractility markers counteracting osteogenic phenotypic switching in vitro, while increasing ectopic SMC calcification.

## 4. Discussion

In this study, we put forward a key role for PRG4 in modulation of SMCs into an osteogenic phenotype, ECM remodeling and atheroprogression with intimal calcification. Using human data, animal models and cell culture, we show that: (i) *PRG4* expression by SMCs appears as an early reaction to vascular remodeling, preceding the formation of macro-calcification; (ii) osteogenic and inflammatory growth factors, high calcium and particularly high phosphate conditions induce *PRG4* expression, regulated by *SMAD3* and *SOX9* transcription factors, which accompanies the osteogenic phenotypic switch of SMCs; (iii) as a feedback loop, PRG4-enriched ECM leads to the recovery of typical SMC markers and migration/proliferation arrest under calcifying conditions.

Our previous discovery of PRG4 in atherosclerosis from a cohort of patients, where plaque calcification was stratified by CTA assessment [4], was confirmed and extended here by more detailed CTA analyses beyond calcification, including quantification of lipid rich necrotic core and overall plaque burden. Combined, we associate plaque expression levels of PRG4 with clinical surrogate markers of advanced atherosclerosis and plaque phenotype [39]. In human samples, during atherogenesis, PRG4 could be detected intracellularly already in adaptive intimal thickenings and xanthomas, while it was abundant in the ECM from the stage of pathological intimal thickening. In early and thin-cap fibroatheromas, PRG4 was found in shoulder regions, especially around neovessels. During atheroprogression, PRG4 was co-localized with cells positive for SOX9 and later also with RUNX2, TRAP and VWF positive cells, confirming our previously reported correlations between PRG4 expression and these transcripts in plaques [4]. SOX9 and RUNX2 are important transcription factors during bone development and homeostasis, controlling chondrocytic and osteoblastic differentiation pathways [49,50]. Both have previously been linked to plaque calcification and reprogramming of SMCs towards osteogenic expression patterns [51,52]. Engagement of VWF^+^ endothelial cells and TRAP^+^ osteoclasts is a key event in endochondral bone formation and remodeling, with PRG4 implicated in this process [15,53], although the role of TRAP in vascular calcification remains debated [54]. While a link between calcification and neovascularization has been shown in aortic valves [55], the findings are not equally clear in carotid plaques. We speculate that the same mechanism may be extended to advanced intimal calcification, where PRG4 was clearly deposited around macro-calcifications and neovessels. Overall, our IHC analysis of human atheroprogression indicated that PRG4 appears to be connected to the formation of calcified ECM by osteoblast-like cells, which can be derived from transdifferentiated SMCs as shown in recent studies using in vivo lineage-tracing models [9]. However, in human lesions in situ, SMA could not be co-localized in the same cells as SOX9 or RUNX2 markers, likely due to the inverse regulatory functions of pro-osteogenic transcription factors and MYOCD. Specifically, direct interaction between SOX9 and MYOCD has been previously reported to mediate osteogenic modulation of SMCs [56].

Nevertheless, PRG4 was detected in regions rich with SMCs, the major cell type responsible for intimal remodeling [22,46]. This relationship was further investigated using two rodent models of intimal hyperplasia, carotid ligation on ApoE^−/−^ mice and rat carotid balloon injury. The carotid ligation model has been associated with an early local reaction via a marked increase in inflammatory cytokines that arise both directly from injured SMCs and endothelial cells, as well as from adhered blood cells and platelets. SMCs not only secrete these cytokines but can also respond to them in an autocrine fashion, leading to further increases in their secretion in a positive feedback loop, SMC activation and neointimal growth after several weeks. In this model, *PRG4* was upregulated and the protein found to be abundant in the neointimal ECM, along with a positive correlation to *SOX9* and *RUNX2*. Similarly, in the rat carotid balloon injury model, we and others have shown that intimal hyperplasia develops in three major stages [57,58], starting with early inflammatory response during the first two days after injury, while, between days two and five, SMCs activate and migrate to colonize the intimal surface [28]. During the next few weeks, neointimal SMCs replicate, but 6–12 weeks after injury, cells become quiescent and regain ultrastructural features typical for a differentiated state [58]. Here, gene expression analyses and immunohistochemistry confirmed an early upregulation of PRG4 and SOX9 during the acute inflammatory phase, which correlated with other osteogenic markers, while both genes were repressed in the late resolution phase after injury. Our results from both of these rodent models indicate that osteogenic programs may be engaged in the response of SMCs to vascular injury, especially elicited by cytokines such as TGFb1 that were also elevated, even without ECM calcification. Interestingly, the subsequent deposition of extracellular PRG4 was associated with the repression of osteogenic pathways and regained expression of typical SMC related genes observed late after injury. Considering the lubricating, immuno-modulating and cyto-protective potential of PRG4 [11,12,59,60,61], our findings suggest that upregulation of *Prg4* could be an early protective reaction by SMCs to tissue stress inflicted by biomechanical forces and atherogenic stimuli. We also show that PRG4 and SOX9 induction preceded the formation of macro-calcification nodules in the mouse model of calcific atherosclerosis [29], supporting the role of PRG4 in early osteogenic modulation during atheroprogression.

In order to functionally and mechanistically explore the role of *PRG4* in intimal remodeling and calcification [4], we exposed human SMCs in vitro to growth factors typically present in the atherosclerotic milieu. We show that *PRG4* expression in SMCs can be induced by various inflammatory and osteogenic growth factors, where TGFb1 exhibited the most prominent effect. Studies in articular cartilage chondrocytes previously revealed common chondroprotective pathways between SOX9 and SMAD3 regulated by TGFb1 signaling, including *PRG4* expression [48,62,63]. Here, using siRNA knockdown experiments upon TGFb1 stimulation, we demonstrated a similar signaling pathway in control of *PRG4* expression in SMCs, where *SMAD3*, activated by TGFb1, is driving *PRG4* induction while *SOX9* takes a downstream role, further stabilizing chondrogenic expression patterns.

Finally, both calcium and phosphate exposure, which have been identified as important regulators of SMC calcification [64,65], led to an increase in *SMAD3*, *SOX9* and *PRG4* levels, with phosphate being a more pronounced *PRG4* inducer. While both high levels of calcium and phosphate have been causally linked to cellular mineral-overload resulting in intimal and medial calcification [66,67], phosphate has also been shown to be a strong independent driver for osteogenic phenotypic transition of SMCs [65]. Together, these results support the connection between an osteogenic switch of SMCs and *PRG4* upregulation under the influence of prevalent stimuli in atherosclerosis. Considering that we previously showed PRG4 upregulation in connection with valvular calcification [17], it is likely that this gene has a broader role in cardiovascular diseases dependent on inflammation and calcification driven ECM remodeling.

Whereas *PRG4* expression has been shown to be important for cell survival (i.e., in chondrocytes) [11,12,13,14,68], the role of endogenous vs. extracellular PRG4 in atherosclerosis is unknown. While we show that the addition of rhPRG4 to SMCs under calcifying conditions led to increased ectopic calcification, gene expression data showed concomitant downregulation of endogenous *PRG4, SMAD3* and *SOX9*, accompanied by preservation of typical SMC markers. The inhibition of SMC migration and proliferation suggests that PRG4 may both influence calcification and SMC phenotype in atherosclerosis. While changes in SOX9 and SMAD3 levels have already been shown to induce osteogenic SMC transformation [64,69,70], our data indicate that, together with a PRG4-enriched matrix, these effects may lead to increased ectopic calcification. Moreover, we speculate that this matrix in turn enables SMCs to restore a gene expression profile resembling a more differentiated phenotype.

### Limitations

Primary human aortic and carotid SMCs at low passages were used in this study. While these cells express the typical markers and have functional features of differentiated SMCs, we cannot exclude that some of the more sensitive markers are already downregulated even at the early stage after cell isolation, contributing to the onset of phenotypic modulation. Furthermore, cells isolated from different donors might show inherently distinct predispositions to calcification and transdifferentiation. We conducted exploratory studies on commercially available primary cells from donors not diagnosed with cardiovascular disease. Additionally, we extended our analysis to cells isolated from biopsies classified as normal aortic wall tissue, from patients undergoing thoracic aneurysm surgery. Nevertheless, it cannot be excluded that results are affected by interpatient variability. With respect to the rat carotid artery injury model, because of the limited amount of tissue available, transcript and histological analyses were performed on different parts of the artery. This could have consequences for data interpretation since the re-endothelialization process in this model is incomplete and leaves the central part of the artery without endothelial coverage. It may be of interest to point out that the warfarin-model of vascular calcification is restricted to certain strains of mice (DBA/2 background, but not the C57BL/6) and has only recently been confirmed to induce intimal plaques in ApoE-deficient mice [29]. When it comes to human studies, BiKE comprises late-stage carotid plaques collected at surgery, and histological classification (i.e., by AHA grading) in this cohort is not performed. Clinical patient assessment is applied instead, and we estimate that all BiKE plaques correspond to AHA grade VI and VII. To analyze PRG4 in relation to human atheroprogression, extending our observations to another vascular bed, we used an independent, worldwide unique cohort of autopsy specimens (Sokrates, Leiden, The Netherlands) graded according to AHA definition. Due to the rareness of these tissues, n could not be further expanded.

## 5. Conclusions

Altogether, our studies position PRG4 as one of the most enriched molecules in highly calcified human carotid plaques [4] and also during intimal remodeling in response to injury. Here, we provide the first functional and mechanistic evidence that PRG4 is of importance for SMC osteogenic transformation via the TGFb1-SMAD3-SOX9 signaling axis. As a component of the ECM, PRG4 is an early signature marker of vascular remodeling, preceding, and likely facilitating, the formation of macro-calcified nodules. Further studies should address whether PRG4 has a similar role in other forms of cardiovascular calcification and whether it has a translational value as a marker of an atherosclerotic plaque phenotype.

## Data Availability

Material and Data pertaining to this manuscript are available from the corresponding author pending reasonable request. Restrictions associated with human biobank protection and personal data GDPR legislation will be respected. The BiKE microarray datasets are available from Gene Expression Omnibus (accession nrs GSE21545 and GSE125771).

## Supplementary Materials

The following are available online at https://www.mdpi.com/article/10.3390/cells10061276/s1, Figure S1: PRG4 knockdown affects expression of SMC contractile markers and addition of PRG4 protein reduces SMC proliferation under TGFβ1 and PDGFB stimulation, Figure S2: Validation of calcification induction in vitro by high calcium and phosphate levels, Figure S3: OxLDL loading induces PRG4 and SOX9 expression in vitro, Figure S4: No significant passive calcification caused by exposure to rhPRG4 protein in the absence of SMCs, Figure S5: RhPRG4 protein stabilizes expression of contractile SMC markers, Table S1: PRG4 plaque mRNA expression is independently correlated to calcification volume.

## Non-Standard Abbreviations and Acronyms

CALC	coronary artery calcification
CALC Vol Prop	calcification volume proportion
ECM	extracellular matrix
HAoSMCs	human aortic smooth muscle cells
HCtSMCs	human carotid smooth muscle cells
LRNC Vol Prop	lipid rich necrotic core volume proportion
rhPRG4	recombinant human proteoglycan 4
TMA	tissue microarray
WTD	western type diet
