# Supplementary material for: Proteoglycan 4 Modulates Osteogenic Smooth Muscle Cell Differentiation during Vascular Remodeling and Intimal Calcification
Source: Cells. 2021 May 21;10(6):1276. doi: 10.3390/cells10061276 (PMC8224064; doi:10.3390/cells10061276)
Supplement: Supplementary file 1 [file cells-10-01276-s001.zip › cells-1216571-supplementary.pdf]

Supplemental Material

Proteoglycan 4 modulates osteogenic smooth muscle cell differentiation during vascular remodeling and intimal calcification

Till Seime[1], Asim C. Akbulut[2], Moritz Lindquist Liljeqvist[1], Antti Siika[1], Hong Jin[1,3], Greg Winski[3], Rick H. van Gorp[2], Eva Karlöf[1], Mariette Lengquist[1], Andrew J. Buckler[1], Malin Kronqvist[1], Olivia J. Waring[4], Jan H.N. Lindeman[5], Erik Biessen[4], Lars Maegdefessel[3,6], Anton Razuvaev[1], Leon J. Schurgers[2,7], Ulf Hedin[1*], Ljubica Matic[1*, #]
(*equal contribution)

Supplementary Figures and Legends

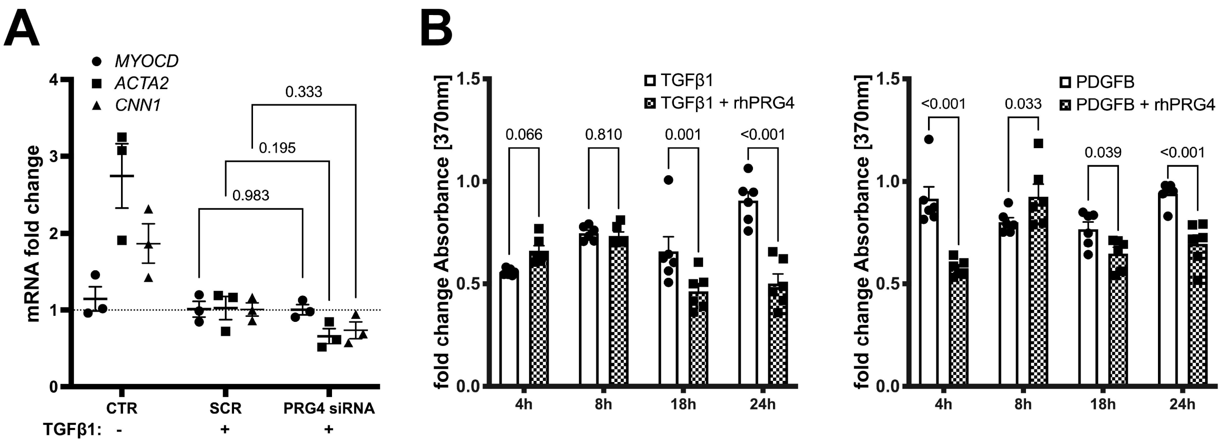

Figure S1. PRG4 knockdown affects expression of SMC contractile markers and addition of PRG4 protein reduces SMC proliferation under TGFβ1 and PDGFB stimulation. (**A**) SiRNA knockdown of *PRG4* downregulated expression of *ACTA2* and *CNN1* in HAoSMCs, however this effect remained non-significant compared to scrambled control. (**B**) RhPRG4 significantly inhibited SMC proliferation under TGFβ1 and PDGFB stimulation. HAoSMCs-human aortic smooth muscle cells. CTR-untreated cells in identical medium and FBS conditions; SCR-scrambled control following TGFβ1; rhPRG4-recombinant human PRG4. Plots show mean with SEM. Statistical difference assessed by 2-way ANOVA, (**A**) n = 3, (**B**) n = 6 experimental replicates.

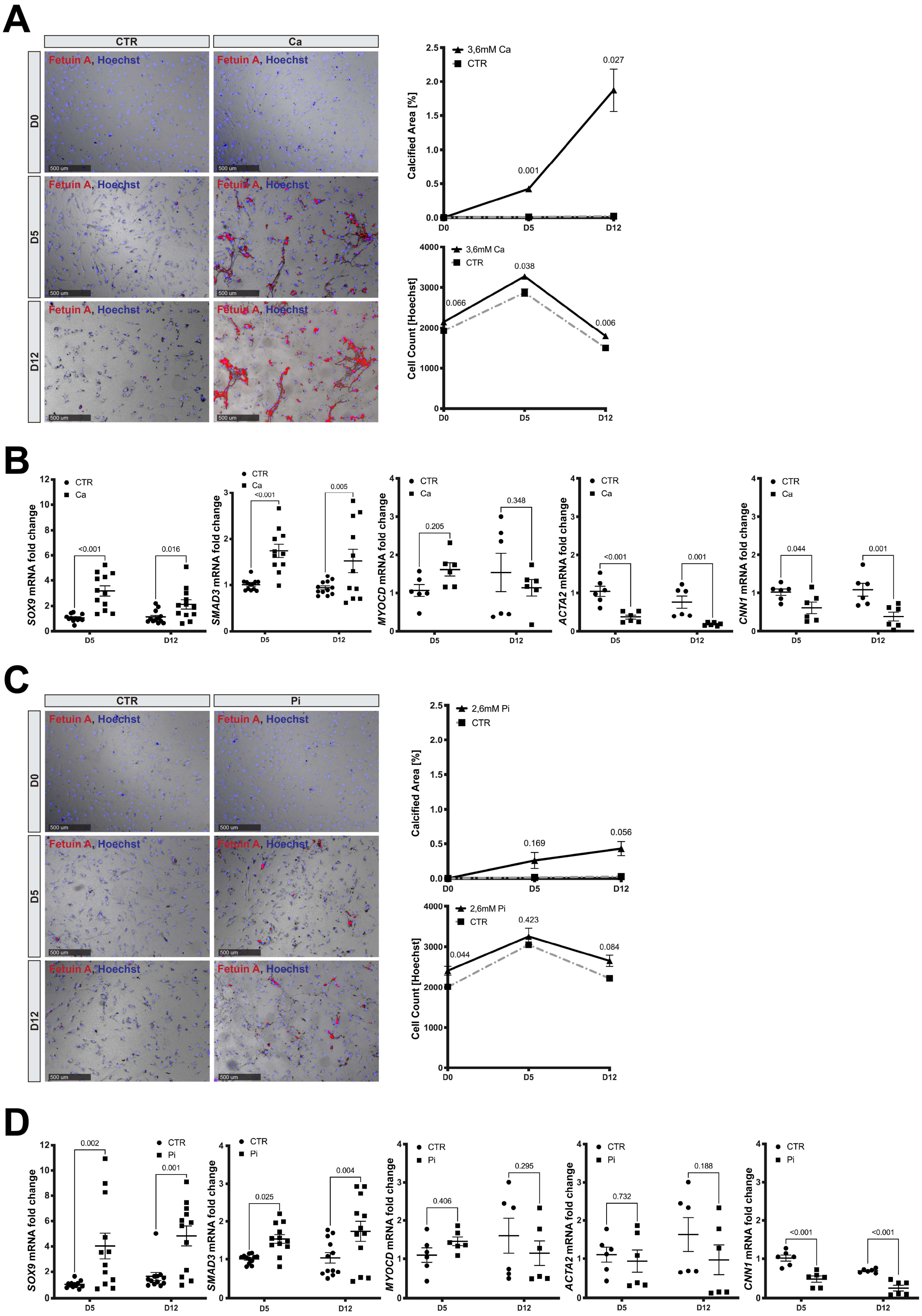

Figure S2. Validation of calcification induction in vitro by high calcium and phosphate levels. (**A**) Stimulation of HA-oSMCs cells with 3.6 mM calcium resulted in extensive sheet-like calcification visualized by an Alexa-fluor 546 coupled Fetuin-A probe. Calcification area (% of total area) was significantly increased already after 5 days (about 50% compared to baseline), while cell numbers assessed by HOECHST stain also increased but only by about 10%. The extent of calcification was up to about 200% after 12 days, while cell numbers stayed roughly unchanged. (**B**) Calcium treatment

significantly elevated levels of *SOX9* and *SMAD3* mRNA and caused a downregulation of typical markers of SMC quiescence and contractility (*MYOCD, ACTA2, CNN1*). (**C**) Exposure to 2.6 mM phosphate resulted in nodular calcifications. Calcification area was significantly increased after 12 days, not affecting cell numbers compared to controls. (**D**) High phosphate stimulation increased *SOX9* and *SMAD3* mRNA expression and caused a downregulation of *MYOCD, ACTA2* and *CNN1*, however not as severe as under high calcium conditions. Images show 4x magnification. CTR-untreated cells in identical medium and FBS conditions; HAoSMCs-human aortic smooth muscle cells. Plots show mean with SEM. Statistical difference assessed by 2-way ANOVA, (**A**, **C**) $n = 3$, (**B**, **D**) $n = 3$ experimental replicates in cells from 3 patients and commercial cells from Lonza.

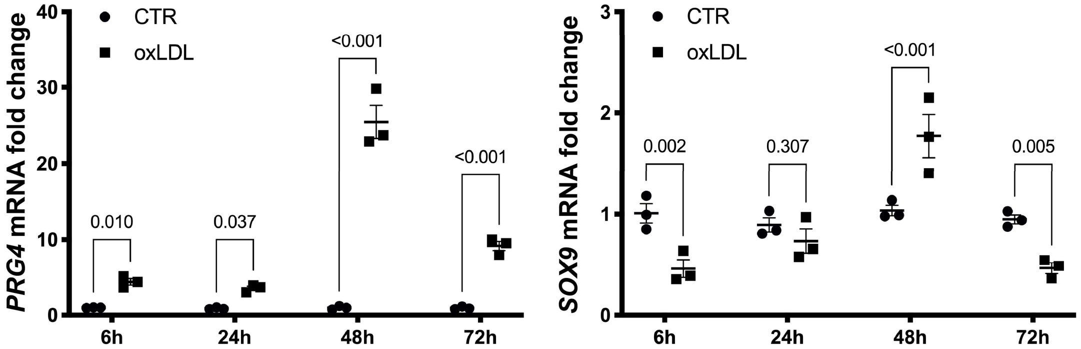

Figure S3. OxLDL loading induces *PRG4* and *SOX9* expression *in vitro*. Loading with 20 µg/ml oxLDL significantly induced *PRG4* in HAoSMCs, especially after 48h. At this time point *PRG4* expression coincided with increased *SOX9* mRNA levels. Plots show mean with SEM. Statistical difference assessed by 2-way ANOVA, $n = 3$ experimental replicates.

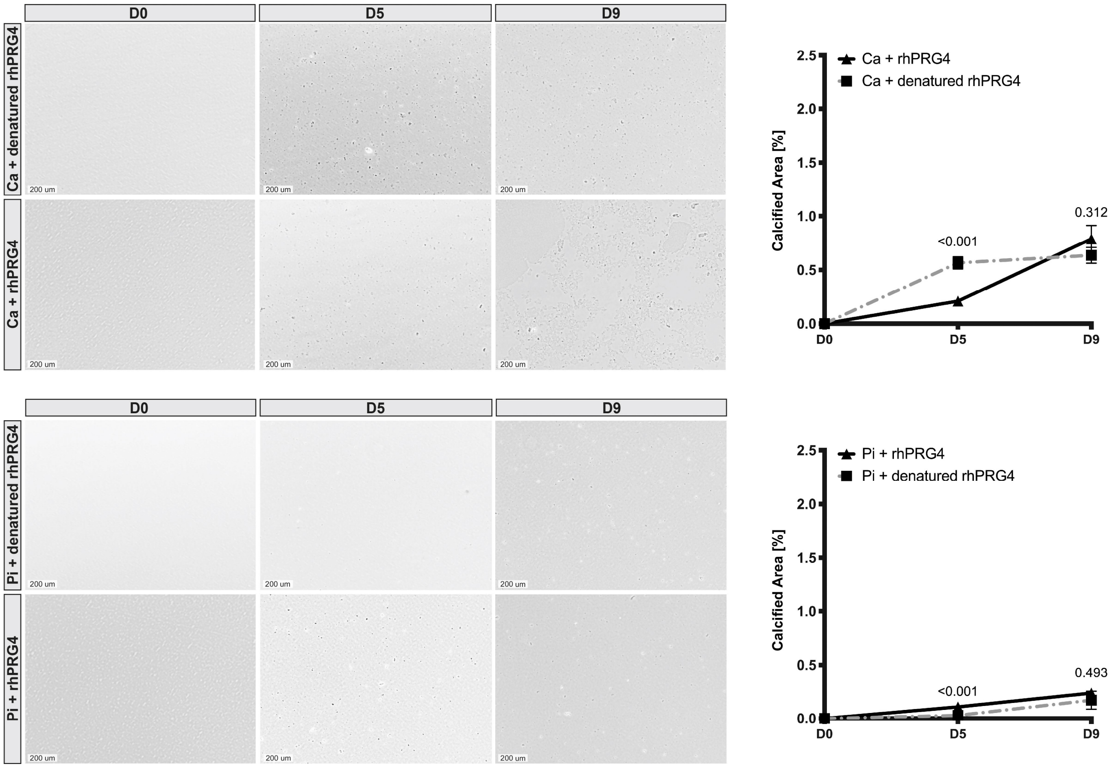

Figure S4. No significant passive calcification caused by exposure to rhPRG4 protein in the absence of SMCs. In the absence of cells rhPRG4 only caused minor passive precipitations below 1% of total area both in combination with high calcium or phosphate conditions. This effect showed no significant difference to the passive effect of heat inactivated rhPRG4. Images show 10x magnification. rhPRG4-recombinant human PRG4 protein. Plots show mean with SEM. Statistical difference assessed by 2-way ANOVA, $n = 3$ experimental replicates.

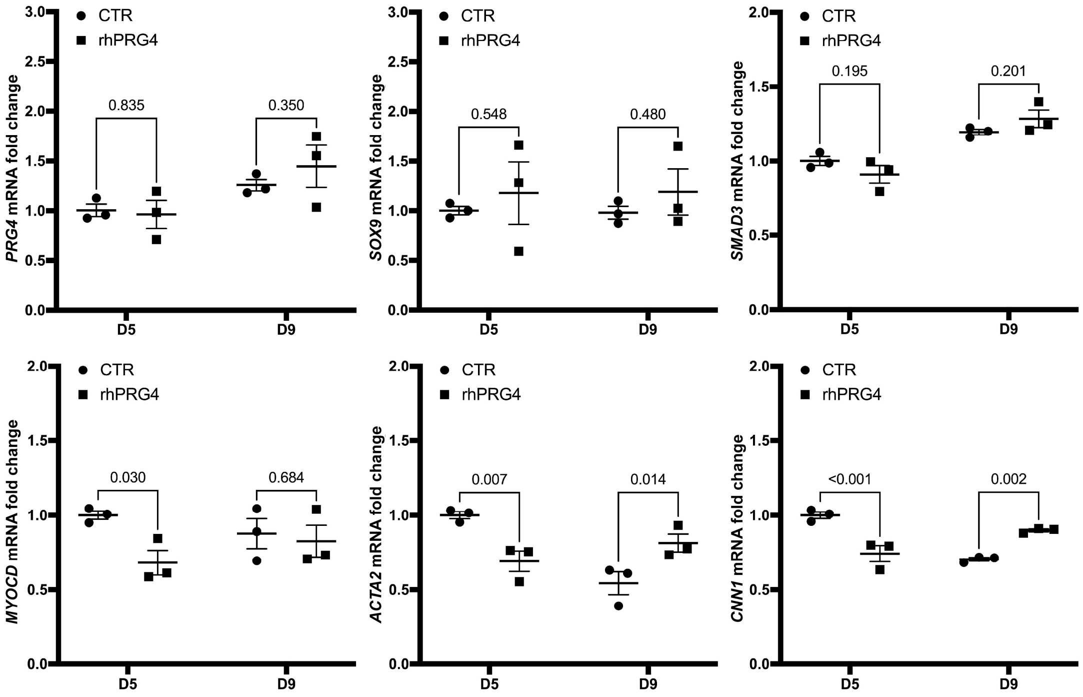

Figure 5. RhPRG4 protein stabilizes expression of contractile SMC markers. Without calcifying conditions, rhPRG4 supplementation had no significant effect on *PRG4*, *SOX9* or *SMAD3* mRNA expression of HAoSMCs. However, after an initial downregulation, typical markers of SMC quiescence and contractility stabilized and remained significantly higher compared to controls after 9 days. Images show 4x magnification. CTR-untreated cells in identical medium and FBS conditions; HAoSMCs-human aortic smooth muscle cells. Plots show mean with SEM. Statistical difference assessed by 2-way ANOVA, $n = 3$.

Table S1. PRG4 plaque mRNA expression is independently correlated to calcification volume. Multiple linear regression analysis of PRG4 mRNA expression with calcification volume (CalcVolProp), lipid rich necrotic core volume (LRN-CVolProp) and plaque burden volume (PlaqueBurdenVolRatio) in late-stage human atherosclerotic plaques.

Variable	Estimate	95% CI (asymptotic)	P value
Intercept	3.74	–0.94 to 8.41	0.11
CALCVolProp [%]	0.07	0.003 to 0.13	0.04
LRNCVolProp [%]	0.005	–0.07 to 0.084	0.90
PlaqueBurdenVolRatio	5.10	–3.80 to 14.00	0.25
R squared=0.35			

Major Resources Table.

Animals (in vivo studies).

Species	Vendor or Source	Background Strain	Sex
ApoE$^{-/-}$ mice	Taconic Biosciences	C57BL/6	male
ApoE$^{-/-}$ mice	Maastricht University	C57BL6/J	male
Sprague-Dawley rats	Charles Rivers	SD	male

Antibodies.

Target antigen	Vendor or Source	Catalog #	Working concentration
PRG4	Sigma	HPA028523	1:25 - 1:50
SMA	DAKO	M0851	1:1500
CD68	DAKO	M0876	1:100
TRAP	LS BIO	LS-C87845	1:50
SOX9	Sigma	AMAB90795	1:200
SOX9	Abcam	Ab26414	1:500
RUNX2	Sigma	AMAB90591	1:200
VWF	DAKO	M0616	1:500

Cultured Cells.

Name	Vendor or Source	Catalog #	Sex (F, M, or unknown)
human carotid smooth muscle cells	Cell Aplications	3514-05A	unknown
human ascending aortic smooth muscle cells	Lonza	CC-2571	M

Data & Code Availability.

Description	Source / Repository	Persistent ID / URL
BiKE microarray dataset	Gene Expression Omnibus	GSE21545

Other.

Description	Vendor or Source	Catalog #
siRNA PRG4	Thermo Fisher	#s19926
siRNA SOX9	Thermo Fisher	#s532695
siRNA SMAD3	Thermo Fisher	#s8401
Taqman assay PRG4	Thermo Fisher	Hs00981633_m1
Taqman assay SOX9	Thermo Fisher	Hs00165814_m1
Taqman assay BMP2	Thermo Fisher	Hs00154192_m1
Taqman assay MYOCD	Thermo Fisher	Hs00538076_m1
Taqman assay ACTA2	Thermo Fisher	Hs00426835_g1
Taqman assay MYH11	Thermo Fisher	Hs00975796_m1
Taqman assay CNN1	Thermo Fisher	Hs00959434_m1
PDGFB	Gibco	#PHG0044
TGFβ1	Sigma	#T7039
IFNγ	R&D Systems	#285-IF-100
IGF1	Sigma	#I3769
IGF2	Sigma	#I2526
